# Comprehensive Review of Polymer Architecture for All-Solid-State Lithium Rechargeable Batteries

**DOI:** 10.3390/ma13112488

**Published:** 2020-05-29

**Authors:** Xuewei Zhang, Jean-Christophe Daigle, Karim Zaghib

**Affiliations:** Center of Excellence in Transportation Electrification and Energy Storage (CETEES), Hydro-Québec, 1806, Lionel-Boulet Blvd., Varennes, QC J3X 1S1, Canada; Zhang.Xuewei@hydro.qc.ca (X.Z.); daigle.jean-christophe@hydro.qc.ca (J.-C.D.)

**Keywords:** polymer, lithium bis(trifluoromethanesulfonyl)imide (LiTFSI), lithium, SPE, poly(ethylene oxide) PEO

## Abstract

Solid-state batteries are an emerging option for next-generation traction batteries because they are safe and have a high energy density. Accordingly, in polymer research, one of the main goals is to achieve solid polymer electrolytes (SPEs) that could be facilely fabricated into any preferred size of thin films with high ionic conductivity as well as favorable mechanical properties. In particular, in the past two decades, many polymer materials of various structures have been applied to improve the performance of SPEs. In this review, the influences of polymer architecture on the physical and electrochemical properties of an SPE in lithium solid polymer batteries are systematically summarized. The discussion mainly focuses on four principal categories: linear, comb-like, hyper-branched, and crosslinked polymers, which have been widely reported in recent investigations as capable of optimizing the balance between mechanical resistance, ionic conductivity, and electrochemical stability. This paper presents new insights into the design and exploration of novel high-performance SPEs for lithium solid polymer batteries.

## 1. Introduction

**Figure 1:**
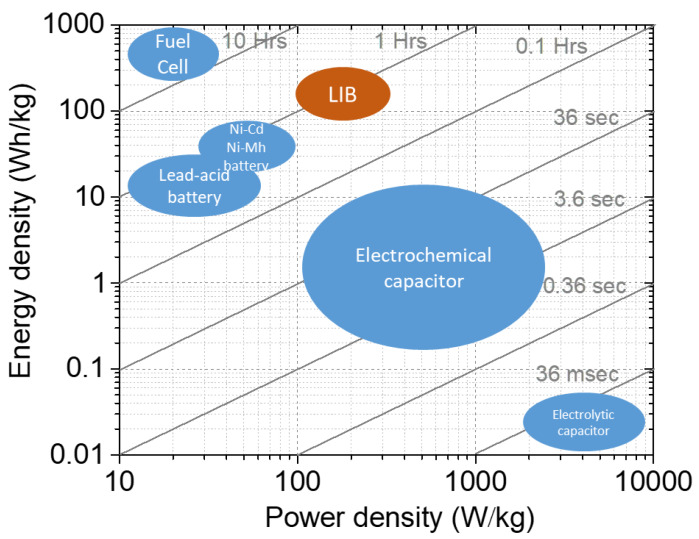
Ragone plot of some energy storage devices.

As a major portable power source, lithium-ion batteries (LIBs) are widely used in consumer electronics as well as electric vehicles (EVs). Because they present a higher power density than conventional secondary batteries such as Ni-Cd batteries, and a higher energy density than capacitors, like shown in Figure 1 [1,2,3,4,5]. Currently, most of the LIBs utilize liquid electrolytes because of their high ionic conductivity at ambient temperature. However, the safety issues of liquid electrolytes, such as the flammability and leakage, are hindering the development of LIBs [6]. The appearance of gelled polymer electrolytes (GPEs), in which liquid solvent is immobilized in a polymer matrix [7], can reduce the risk of leakage, but not the flammability. An SPE is defined as an ion-conductive phase composed of salt and salt-solvating macromolecules [8,9]. Compared with conventional lithium-ion batteries with a liquid electrolyte, using SPEs can enhance the energy density and safety of batteries [10,11]. Ever since PEO/alkali salt complex as a polymer electrolyte in lithium batteries was first reported in 1978 [12], the use of this type of electrolyte has captured the interest of researchers worldwide. In the 1980s, an exploration period for SPEs, the investigations were focused on ion transport mechanism and ionic conductivity enhancement as well as the production of lithium secondary batteries. Hydro-Québec and two French laboratories reported the first 3 V lithium secondary battery with a PEO electrolyte in 1985 [13]. Chatani et al. [14] published the first full crystallographic determination of a PEO/salt complex in 1987. The 1990s and 2000s represented a fast development period of LIBs, including electrode and electrolyte materials [15]. In 1991, the successful commercialization of LIB by Sony initiated a steep increase in the production of portable electronics, which accelerated the development of high energy density LIBs. In the same period, lithium-polymer battery technology was also developed for electric vehicle applications. In 1998, 3M (Rogers, AR, USA), Hydro-Québec and Argonne National Laboratories developed a lithium polymer battery of 119 Ah for EVs [16]. In recent years, the emergence of numerous new host polymers and salts has also given impetus to the development of solid polymer electrolytes.

**Figure 2:**
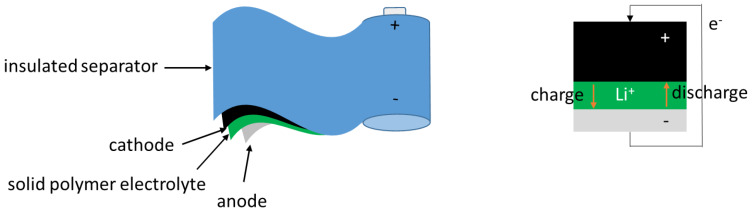
Illustration of all solid-state lithium polymer batteries (ASSLPB) composition.

In view of the current global climate situation, it is urgent to develop zero-emission power sources to reduce greenhouse effects. The first candidates for such power sources are all solid-state lithium polymer batteries (ASSLPBs). This is because they exhibit balanced characteristics of safety, energy density, power density, and cost compared with other batteries [17,18]. In 2011, the first commercial EV (Bluecar^®^) equipped ASSLPB as a power source appeared in Paris [19]. Today, the widespread diffusion of EVs all over the world irritates the battery technology improvement. Accordingly, to achieve high power performance, this research is focused on the development of battery materials. In their present state, ASSLPBs generally consist of a metallic lithium anode, a lithium iron phosphate (LFP) or nickel-manganese-cobalt (NMC) cathode, an SPE, and an insulated separator, as illustrated in Figure 2. In the SPE, the lithium ions are set in motion when the battery is charged or discharged. Compared with ceramic electrolytes, SPEs exhibit a lower interfacial impedance with electrodes because of their better flexibility and interfacial adhesion. Currently, SPEs are not only employed in lithium batteries but also in other energy accumulators, such as fuel cells [20], dye-sensitized solar cells [21], and supercapacitors [22]. Despite the many advantages that SPEs afford over liquid and ceramic electrolytes, they also exhibit certain disadvantages, such as lower ionic conductivity at low temperatures (<50 °C) and poor electrochemical stability at high voltage levels (>4.5 V). To overcome these limitations, considerable efforts have therefore been devoted to their investigation. An ideal SPE should be easy to fabricate and inexpensive, as well as possess high ionic conductivity at a wide temperature range, high chemical and electrical stability, and good mechanical strength and flexibility.

Many excellent reviews have summarized the development of SPEs, including PEO-based SPEs [23,24,25], poly(ionic liquid) (PIL) SPEs [26,27], composite SPEs [28,29], single lithium-ion conducting SPEs [30], and SPE additives and salts [31,32]. This paper focuses on the influence of polymer architecture on the physical and electrochemical properties of SPEs in ASSLPBs, which is yet to be reviewed. To optimize the balance between mechanical resistance, ionic conductivity, and electrochemical stability, four principal categories, which have been widely elaborated in recent investigations, are examined: linear, comb-like, hyper-branched, and crosslinked polymers. In view of the various ion transport mechanisms, the discussion centers on two types of electrolytes: PEO-based and PIL SPEs. It is assumed that this review will provide new insights into the design of novel high-performance SPEs for ASSLPBs.

## 2. Theoretical Background

Before polymer architecture is discussed, the ion transport mechanism of SPEs is briefly reviewed herein. As known, most polymers are recognized as poor ionic conductors. The ionic conductivity of a polymer electrolyte generally emanates from the contributions of all free ions of dissolved salts or polymeric salts, as expressed by:

(1) $$\sigma =\sum^{}n_{i}q_{i}\mu_{i}$$

where *n_i_* is the ion concentration (number of ions per unit volume); *q_i_* is the ion charge; *μ_i_* is the ion mobility (average ion velocity), which can be further described by the following Einstein relationship:

(2) $$\mu =\frac{Dq}{k_{B}T}$$

where *D* is the diffusion constant; *k_B_* is Boltzmann constant; *T* is the absolute temperature. The ionic conductivity of a given polymer electrolyte is therefore mainly associated with ion concentration and temperature. 

Solid polymer electrolytes can be categorized into salt-polymer complex electrolytes and single-ion polymer electrolytes (polymeric salts) according to their composition. In the former, lithium salts are dissolved in polymer matrices containing atoms or functional groups that are capable of coordinating with cations. It is found that only oxygen, nitrogen, and sulfur atoms can form the coordination bond with lithium ions [33]. The stability of lithium complexes decreases in the following order: O > N > S. Single-ion polymer electrolytes typically consist of polymerized anions and mobile lithium cations or an anion acceptor [34]. In fact, both this type of polymer electrolytes and PILs can be considered as polyelectrolytes in which polymer chains bear dissociating groups in their repeat units [35].

**Figure 3:**
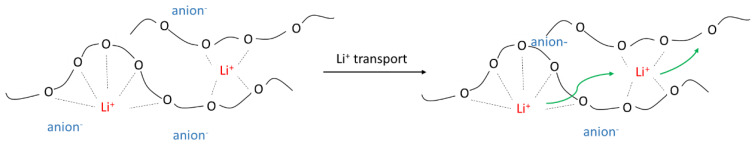
Ion transport mechanism in non-ionic polymer.

When a non-ionic polymer is used as an ion-solvating medium in salt-polymer complex electrolytes (e.g., PEO), lithium cations transfer by hopping between any two adjacent coordinating sites located in the intra-chain and inter-chain in an electric field, as shown in Figure 3.

The dynamics of cations is linked to the polymer segmental motion in the amorphous state above the glass transition temperature (*T_g_*) of the polymer. No evident ion motion has been observed in the crystalline and amorphous sections at temperatures below *T_g_* [36,37]. In this case, the relationship between temperature and ion conductivity can be described by the non-linear Vogel–Fulcher–Tammann (VFT) equation:

(3) $$\sigma =\sigma_{0}exp\left(-\frac{B}{T-T_{0}}\right)$$

where *σ*_0_ is the pre-exponential parameter and *B* is the pseudo-activation energy; *T*_0_ is an empirically determined temperature (10–50 °C) that is below *T_g_*.

In another case, the Li^+^ transport is less dependent on the segmental motion. The conduction mechanism follows Arrhenius model:

(4) $$\sigma =\sigma_{0}exp\left(-\frac{E_{0}}{k_{B}T}\right)$$

where *E*_0_ is the activation energy and *k_B_* is the Boltzmann constant. The relationship between ion conductivity and 1/*T* is linear, and the activation energy *E*_0_ can be determined by finding the slop of Arrhenius plots.

Compared to non-ionic polymers, ionic hosts, such as PILs, have a higher dielectric constant and electrochemical stability; most PILs have a measure of ion conductivity. In ionic liquid electrolytes, the Li^+^ cation transport occurs by anion exchange with its adjacent coordinating sites during Li^+^ movement via electrostatic interaction [38], which is similar to vehicular mechanism [39]. With regard to PILs, most studies on ion transport focus on pure polymer without lithium salt addition [40]; however, the lithium cation transport mechanism in PILs has not been reported.

As mentioned, the cations and anions in salt-polymer complex electrolytes are all mobile. This usually reduces the transference number, which is defined as the ratio of the electric current derived from the cation to the total electric current, thereby leading to concentration polarization. In order to increase this number, the counterpart of lithium cation can be attached to the macromolecular chains by a covalent bond or an anion acceptor. Computer simulations and experimental results demonstrate that the cation transport in this case also occurs through ion hopping among anion sites and considerably depends on the rotation of polymer segments. A higher ionic conductivity can be obtained for a single-ion polymer electrolyte with a lower *T_g_*, such as salt-polymer electrolytes. Despite the high cation transference number in a single-ion polymer electrolyte, however, its ionic conductivity is lower than that of salt-polymer electrolyte because of the insufficient dissociation of Li^+^ ions in such electrolytes.

Overall, regardless of the electrolyte system, the ion transport is mainly linked to polymer segmental mobility, i.e., the *T_g_* of polymer host. Another important factor that varies with the electrolyte architecture is its mechanical strength. When metallic lithium is used as the battery anode, high tensile modulus electrolytes can inhibit the nucleation and growth of lithium dendrites which often cause a battery short-circuit [41,42]. In the following sections, the architecture of the polymer host used in SPEs and its effect on ion mobility, as well as its mechanical properties and electrochemical stability, are summarized.

## 3. PEO-Based SPEs

### 3.1. Linear Polymer

The simplest polymer structure is a linear chain, and initial investigations of SPEs started with a linear PEO. Wright et al. first discovered the ionic conductivity of PEO/alkali salts in 1973 [43]. Five years later, M. Armand proposed the use of this complex as an electrolyte in secondary lithium batteries [10]. In the past 40 years, it has been demonstrated that PEO, which is the most frequently studied polymer host, has good ionic conductivity in an amorphous state and electrochemical stability. It also has an excellent compatibility with other organic or inorganic materials, which is crucial for a polymer to be regarded as the best candidate for SPEs. The advantages and disadvantages of PEO-based electrolytes have already been reported by certain articles [23,24]; hence, these are not repeated here. 

In addition to PEO, polymers that contain oxygen or nitrogen atoms (e.g., polyethers, polycarbonates, polyesters, and polynitriles) as solvating hosts of lithium salts have been investigated. However, the degradation of polycarbonates and polyesters [44] and the low ionic conductivity of lithium cation in polynitriles [45] limit their further application.

Although other polyethers, such as poly(propylene oxide) [46,47], polytetrahydrofuran [48], and poly(1,3-dioxolane) [49,50], have also exhibited physical and electrochemical properties similar to those of PEO, these have not been widely investigated because of the dearth of products with high molecular weights. 

At present, as the demands of the battery market continue to increase, there remain three main problems in PEO-based SPEs that should be resolved. These include low ionic conductivity at low temperatures, low transference number, and relatively narrow electrochemical window when a high-voltage cathode is used. To overcome the foregoing, various strategies have been conceived, including the addition of organic plasticizers [51], inorganic fillers [52], natural fibers [53], and ceramic particles [54] to SPEs. The use of PEO-based polymer blends and copolymers has also been explored, and a recent review has summarized the benefits of employing PEO-based block copolymer electrolytes for lithium batteries [55]. The incorporation of different polymer segments can enhance not only the ionic conductivity but also the mechanical properties of PEO. Furthermore, copolymers with a single-lithium ion block have been observed to exhibit a higher transference number.

Another effective approach that affects the electrochemical properties of PEO-based electrolytes is modifying the polymer host architecture. Three structures are discussed in the succeeding paragraphs.

### 3.2. Comb-Like Polymer

Typically, the operational temperature of linear PEO-based SPEs is approximately 80 °C, which exceeds the PEO melting temperature. Under this condition, the PEO’s mechanical resistance is considerably inadequate to alleviate the growth of lithium metal dendrites during cycling [56]. Based on this, many comb-like polymers with side oligo(ethylene oxide) chains, acting as lithium cation coordinating sites, were synthesized, and the influence of the main polymer chain structure and grafting degree on ionic conductivity and mechanical strength has been investigated. Table 1 summarizes the common main polymer backbone and some of the electrochemical results.

**Figure 4:**
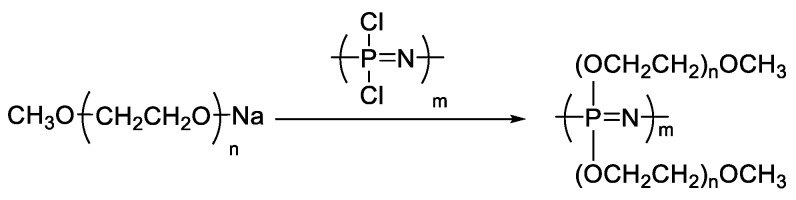
Synthesis of oxyethylene-grafted polyphosphazenes (MEEPs).

Polyphosphazene [57,58] and polysiloxane are generally used as the main polymer chains for oxyethylene-grafted polymer electrolytes. The benefits afforded by these main chains result from their thermal stability, high dielectric constant, and flexibility. Oxyethylene-grafted polyphosphazenes (MEEPs) are usually synthesized with alkoxide-end oligo(ethylene oxide) and poly(chlorophosphazene) (Figure 4). In literature, however, only extremely short grafted side chains are reported [59,60]. The MEEPs are amorphous with a *T_g_* less than −60 °C [61], indicating good segmental mobility. Consequently, MEEP-based SPEs exhibit a considerably higher conductivity than PEOs at room temperature [62]. However, MEEPs undergo viscous flow under pressure because of the lack of dimensional stability and mechanical resistance.

Polymers, being considerably similar to MEEPs (with polysiloxane as backbone and side PEO chains), are also amorphous with a low *T_g_* value of less than −60 °C. In view of this, polymers also have a good ionic conductivity at room temperature but insufficient mechanical strength [63]; hence, polysiloxane-based SPEs usually have a crosslinking structure. Their mechanical and electrochemical properties are discussed in the section on crosslinked polymers. Borodin et al. presented a report on the mechanism of lithium cation transport in an all-ethylene oxide (EO) comb-like polymer in which a considerably flexible main chain was used [64]. Their study clearly illustrates the occurrence of ion transport in the main and side chains—Li^+^ cations have the highest and lowest probabilities of being coordinated by EOs near the polyepoxide ether polymer backbone and at the side chain ends, respectively. The most mobile Li^+^ cations hop from a side chain to another without being complexed by the backbone.

**Figure 5:**
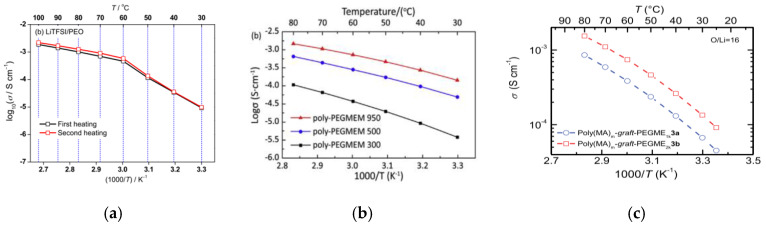
Temperature dependence of ionic conductivity of (**a**) LiTFSI/PEO polymer electrolytes with a molar ratio EO/Li^+^ = 20 [66]; (**b**) LiTFSI/poly-PEGMEM with a molar ratio EO/Li^+^ = 18 [65]; (**c**) LiTFSI/polymethacrylate-*graft*-poly(ethylene glycol) monomethyl ether (Poly(MA)-*g*-PEGME) with a molar ratio EO/Li^+^ = 16 [67]. Reproduced with permission from [65]. Copyright 2019, The Chinese Ceramic Society; [66]. Copyright 2014, Elsevier E. V.; [67]. Copyright 2019, American Chemical Society.

Compared with flexible backbones, a methacrylate polymer main chain is considerably stronger. Poly(ethylene glycol) methacrylate (PEGMA) is the most widely used monomer in the preparation of comb-like polymers with ethylene oxide-containing side groups. The PEGMA homopolymer is amorphous and has a good mechanical resistance. Recently, Yao et al. reported a high-conductivity SPE based on poly(ethylene glycol) methyl ether methacrylate (PEGMEM) [65]. Three comb-like polymer-bearing side poly(ethylene glycol) (PEG) chains with different molecular weights are prepared by ultraviolet (UV) polymerization. Optimal electrochemical results are obtained for the SPE with LiTFSI salt and a 950 g∙mol^−1^ PEGMEM. The SPE exhibits a high conductivity of 1.44 × 10^−4^ S∙cm^−1^ at 30 °C with EO/Li^+^ = 18:1. In a previous report, Zhang et al. studied the physicochemical and electrochemical behaviors of linear PEO in the presence of lithium bis(fluorosulfonyl)imide (LiFSI) and LiTFSI salts [66]. The SPE membranes prepared by solution casting with PEO/LiFSI and PEO/LiTFSI at an EO/Li^+^ = 20:1 exhibit crystallinities of 44% and 47%, respectively, resulting in a decrease in the conductivity of SPEs at 60 °C. A comparison of ionic conductivity as a function of temperature between linear and comb-like polymers is shown in Figure 5. Ignoring the slight difference in Li^+^ concentration, the conductivities in both cases are similar, i.e., above 60 °C. The SPE with a comb-like PEO host, however, exhibits a better conductivity at a lower temperature. Moreover, they all show an electrochemical window (>5 V) and a low transference number (<0.3). In another investigation based on a PEG-grafted SPE, Thelakkat et al. revealed the influence of PEG content on the ionic conductivity [67]. With an EO/Li^+^ = 16:1, the ionic conductivity increases with the side chain length because of the increasing amount of dissolved Li^+^; this observation is similar to the results reported by Yao. However, the anodic breakdown potential is found to be ∼3.5 V vs. Li/Li^+^ because of the electrolyte decomposition by oxidation; this value is lower than that described above. This is probably because of the difference in experimental conditions under which they have been obtained. 

In addition to the methacrylate polymer backbone, other rigid polymers are also used as the main chain. Song et al. reported the effects of grafting degree and side chain lengths on the ionic conductivity using an acrylonitrile-butadiene copolymer backbone with low molecular weight side PEO chains [68]. They found that its degree of crystallinity is significantly reduced, and the mechanical strength is significantly improved compared with those of a linear PEO. The conductivity varies with the side chain length and grafting degree. The maximum conductivity exceeds 10^−5^ S·cm^−1^ at room temperature. Using ring-opening metathesis polymerization, a PEO-grafted polynorbornene with different side chain lengths is synthesized [67]. It exhibits a lower conductivity than a methacrylate backbone polymer because its non-conductive hydrocarbon content in the main chain is higher; similar results are obtained for comb-like PEO−mimetic polypeptoids [69]. In a poly(ether ether ketone)-based comb-like copolymer with short side PEO chains [70], it is also observed that the non-conductive backbone only affects its mechanical resistance, rather than significantly improving its electrochemical properties. In the foregoing examples, only short side chains are grafted. Zardalidis et al. studied the physicochemical and electrochemical properties of poly(hydroxylstyrene) with long side PEO chains [71]. The copolymer exhibits a similar electrochemical behavior as the linear PEO because of the crystallization of PEO chains but a better mechanical strength. Because of the incorporation of rigid main chain, the highest storage modulus (*G*’) obtained at 80 °C reaches 10^8^ Pa, much better than that of linear PEO (*M*_w_ = 22 kg·mol^−1^) at the same temperature [72]. It is also found that *G*’ decreases with the salt concentration. In addition to side PEO chains, Aldalur et al. successfully grafted other ethylene oxide-containing polymers, such as Jeffamine^®^ (diamine-poly(oxyethylene-*co*-oxypropylene)), to a main poly(ethylene-*alt*-maleic anhydride) chain [73,74]. The degree of crystallinity of this polymer decreases with the side chain length similar to that of the side PEO chain copolymer. The ionic conductivity (4.5 × 10^−5^ S∙cm^−1^) of SPE with Jeffamine-based matrix at room temperature is considerably better than that with a linear PEO. 

In conclusion, PEO-based comb-like polymers usually comprised of short PEO chains and a hard backbone, which enhances mechanical strength. The SPEs that constitute such a polymer demonstrate a higher ionic conductivity and a better mechanical resistance compared with linear PEO-based SPEs; however, they exhibit similar transference numbers (< 0.5). The electrochemical stability of these materials is overestimated because it is usually measured by cyclic voltammetry using a symmetric cell with smooth metallic electrodes, which is different from real electrode composition. On the other hand, many poly(ethylene oxide) methacrylate (PEOMA)-based block copolymers with an amorphous block, such as polystyrene [76], poly(n-butyl methacrylate) [77], and poly(methyl methacrylate) [78], are synthesized. The derived results are similar to those of the above mentioned comb-like polymers. In contrast, combining a single-ion transport block with a PEOMA block can improve not only its mechanical properties but also increase its transference number. The single-ion conducting copolymer, i.e., lithium poly[(4-styrenesulfonyl) (trifluoromethanesulfonyl)imide-*co*-methoxy-polyethylene glycol acrylate], reported by Zhou et al. [79] exhibits the highest ionic electrolyte conductivities of 7.6 × 10^−6^ S∙cm^−1^ at 25 °C and 10^−4^ S∙cm^−1^ at 60 °C with an EO/Li^+^ = 20.5/1; the transference number (>0.9) approaches unity. In another report [80], a single ion-conducting copolymer composed of poly(lithium 1-[3-(methacryloyloxy)propylsulfonyl]-1-(trifluoromethylsulfonyl)imide) and poly(ethylene glycol) methyl ether methacrylate blocks exhibits similar conductivities of 2.3 × 10^−6^ S∙cm^−1^ at 25 °C and 1.2 × 10^−5^ S∙cm^−1^ at 55 °C with an EO/Li^+^ = 36/1; the transference number is 0.83.

### 3.3. Hyper-Branched Polymer

Different from two-dimensional block copolymers or comb-like copolymer electrolytes, hyper-branched polymers have a globular structure with mobile polymer branches. This type of structure can also suppress PEO crystallization and improve the ionic conductivity and mechanical strength of SPEs. In 2009, Niitani et al. designed a star-shaped copolymer electrolyte with a hyper-branched polystyrene core and poly(poly(ethylene glycol) methyl ether methacrylate) (PPEGMA) arms [81]. Transmission electron microscopy (TEM) and atomic force microscopy (AFM) show that this copolymer forms a well-ordered spherical micro-phase separation structure, in which the star polymers are systematically ordered to form the PPEGMA continuous phase. As a result of the polymer’s unique morphology, the SPEs that contain lithium bis(pentafluoroethanesulfonyl)imide salts exhibit high ionic conductivities, i.e., 10^−4^ S∙cm^−1^ at 30 °C and 10^−5^ S cm^−1^ at 5 °C with EO/Li^+^ = 33/1. Another star-shaped copolymer based on PS and PPEGMA is synthesized by atom transfer radical polymerization [82]. The ionic conductivity increases with the chain length of PEGMA and reaches 8 × 10^−5^ S∙cm^−1^ at 25 °C with EO/Li^+^ = 30/1. 

**Figure 6:**
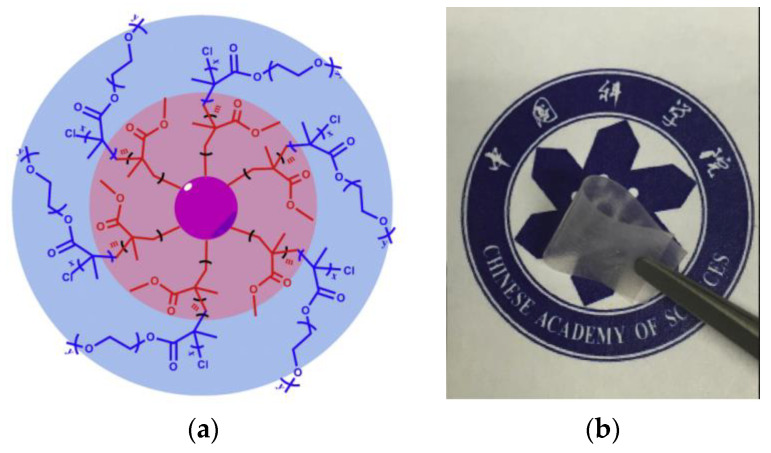
Structure of hyper-branched star polymer HBPS-(PMMA- *b*-PPEGMA)_x_ (**a**) and photograph of HBPS-(PMMA- *b*-PPEGMA)_46_/LiTFSI electrolyte film (**b**) [83]. Reproduced with permission from [83]. Copyright 2016, Elsevier E. V.

Recently, Zhang et al. synthesized a similar core–shell hyper-branched copolymer [83] with hyper-branched polystyrene (HBPS) as core and poly(methyl methacrylate)-*block*-poly(poly(ethylene glycol) methyl ether methacrylate) (PMMA-*b*-PPEGMA) as arms (Figure 6). The ionic conductivities of the polymer electrolyte composed of HBPS-(PMMA-*b*-PPEGMA)_x_ and LiTFSI are 8.3 × 10^−5^ S∙cm^−1^ at 30 °C (PPEGMA content: 83.7%) and 2.0 × 10^−4^ S∙cm^−1^ at 80 °C (PPEGMA content: 51.6%) with a transference number of up to 0.31. Furthermore, the electrolyte exhibited good interfacial compatibility in a symmetric Li/SPE/Li cell. Subsequently, the authors studied the electrochemical behaviors of the composite polymer electrolyte with HBPS-(PMMA-*b*-PPEGMA)_30_ (PPEGMA content: 69.56%), ionic liquid, and LiTFSI [84]. In the presence of ionic liquid, the electrolytes exhibited better ionic conductivities but a considerably lower transference number.

**Figure 7:**
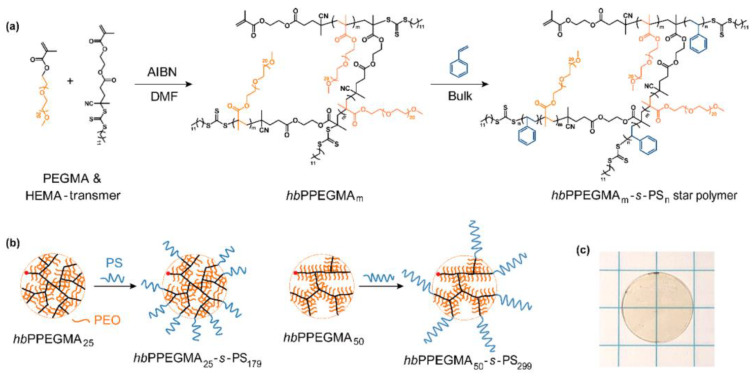
Synthesis of hbPPEGMA_m_-*s*-PS_n_ hyperstar polymers; (**a**) synthetic route; (**b**) schematic of hyper-branched hbPPEGMA_m_ and hbPPEGMA_m_-*s*-PS_n_ polymers with different average chain lengths of branched PEO (m) and linear PS (n); (**c**) digital photograph of hbPPEGMA_50_-*s*-PS_299_ SPE membrane [85]. Reproduced with permission from [85]. Copyright 2019, American Chemical Society.

In contrast to the hard core–soft arm structure, Yao et al. reported a hyper-branched polymer structure with the PEO as star core and the linear polystyrene (PS) as arms (hyper-branched PPEGMA_m_-*s*-PS_n_ (hbPPEGMA_m_-*s*-PS_n_)), as illustrated in Figure 7 [85]. The authors found that the self-assembly of rigid PS arms during phase separation increases the copolymer’s mechanical strength. The highest storage modulus reaches 1.4 MPa for hbPPEGMA_25_-*b*-PS_179_ at 60 °C. The PEGMA chain length affects its segmental motion in the core. The room-temperature ionic conductivities of hbPPEGMA_m_-*s*-PS_n_ SPEs are 5−16 times higher than those of PEO_20k_ and PEO_35k_ SPEs and 2−7 times that of the linear PPEGMA_50_-*b*-PS_179_ SPE. An all-solid-state Li/LiFePO4 battery based on the hyper-branched SPE achieves a stable capacity of 142 mAh∙g^−1^ at 0.2 C.

The branched PEO chains of comb-like and hyper-branched polymer electrolytes are both mobile, and it is this similarity that contributes to the ion transport. In a network structure, crosslinked polymer chains can effectively suppress PEO crystallization, increase thermal and mechanical stabilities, and mitigate the growth of lithium dendrites.

### 3.4. Crosslinked Polymer

In network electrolytes formed by linear PEO chains or corresponding block copolymers without a liquid additive, the ionic conductivity depends on the crosslinking degree and chain length between adjacent junction points [86,87]. Moreover, the ionic conduction mechanism also depends on the mesh size of polymer networks [88]. When this size is small enough, the Li^+^ transport follows Arrhenius model rather than VFT model. In this case, ions jump to the nearest vacant sites. In contrast, when comb-like copolymers with side PEO chains are crosslinked, the PEO chain mobility is better than that of the linear counterpart, resulting in a better ion transport. The network’s mechanical strength varies with the backbone structure and crosslinking degree. H. Kawakami et al. found that linear PEG-based crosslinked network exhibits a much higher stress modulus (6.84 MPa) than PEO (0.55 MPa) with similar molecular mass to the network precursor at ambient temperature [86]. As mentioned, when polysiloxane or polyphosphazene is used as the backbone with the side PEO chain, the network is more flexible than the methacrylate or styrene backbone. The ionic conductivity is mainly tuned by the PEO segment content and mobility as well as the amount of liquid additives introduced. The crosslinking structure is usually obtained through physical means (e.g., micro-phase separation, H-bonding and chain entanglement) or chemical techniques (e.g., copolymerization with a difunctional or multifunctional crosslinker). 

**Figure 8:**
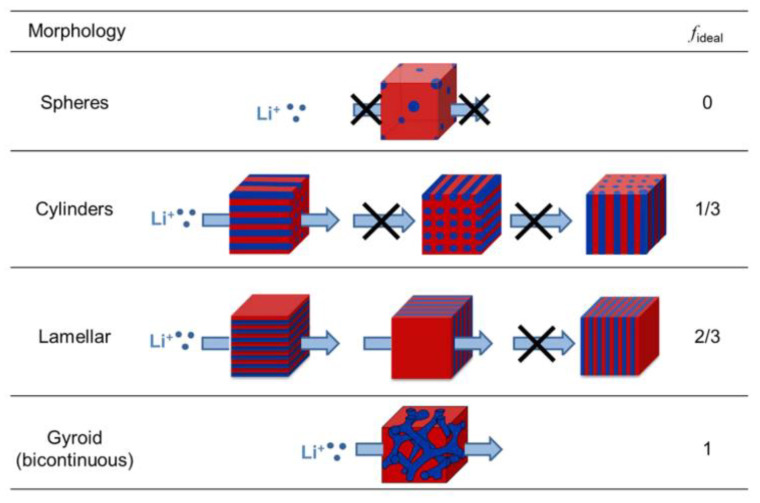
Dependence of ideal morphology factor (*f*_ideal_) on morphology. The blue and red regions represent conducting and non-conducting micro-phases, respectively [93]. Reproduced with permission from [93]. Copyright 2015, American Chemical Society.

Numerous PEO-based block copolymers can form an ion-conducting pathway during the micro-phase separation [89,90,91,92]. Depending on the nature and length ratio of blocks, micro-phases with different morphologies are formed by the copolymer self-assembly. Balsara et al. studied the relationship between ion transport and morphology and the effect of nanoparticle addition on the micro-phase transition [93]. Figure 8 shows the dependence of the ideal morphology factor, *f*_ideal_, on the conductive phase morphology; a bicontinuous morphology provides the best ion transport. The authors also observed that the addition of 2 wt% polyethylene glycol polyhedral oligomeric silsesquioxane (PEO-POSS) nanoparticles into the PS-*b*-PEO copolymer causes a lamellar-to-bicontinuous phase transition, leading to a remarkable increase in the ionic conductivity. This type of phase transition that induces the increase in ionic conductivity is also observed in a PEO/PS/PS-*b*-PEO ternary system [94]. The total homopolymer volume fraction varies from 0 to 0.70, and the ratio, r = [Li^+^]/[EO], is maintained at 0.06. It is found that the increase in conductivity through the order−disorder transition is most probably caused by the elimination of grain boundaries. In either the disordered or ordered state, the conductivity decreases as the total amount of homopolymer increases.

**Figure 9:**
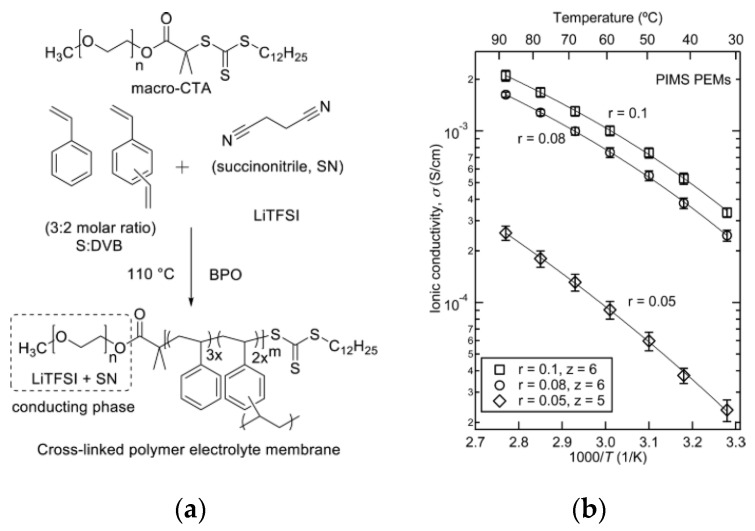
Synthesis route of polymer electrolyte membrane (**a**) and ionic conductivity of membranes with varying LiTFSI contents (**b**) [95]. Reproduced with permission from [95]. Copyright 2017, Elsevier B.V.

Using the same approach, Chopade et al. synthesized a PEO-*b*-PS-based polymer electrolyte through polymerization-induced micro-phase separation (Figure 9) [95]. In this material, the non-conductive phase is chemically crosslinked, thus yielding a higher mechanical strength. The addition of succinonitrile plasticizer allows the electrolyte to reach an excellent conductivity that even exceeds 10^−4^ S∙cm^−1^ at room temperature.

Recently, Wu et al. reported a self-healing solid polymer electrolyte (SHSPE) that is crosslinked by the dynamic intermolecular hydrogen bond between urea and ester groups located in the PEO chains [96]. The authors indicated that the inter-chain hydrogen bond and attractive Coulombic forces among the ions in the lithium salt facilitate fast self-healing. This means that the self-healing rate is considerably affected by the lithium salt concentration. As a result of the fast self-healing capability, a high electrolyte mechanical stability is enabled after damage. The optimized SHSPE with high ionic conductivity (1.9 × 10^−4^ S∙cm^−1^), low electronic conductivity (1.87 × 10^−8^ S·cm^−1^), and high Li-ion transference number (0.44) at room temperature is stable up to 5 V (vs. Li^+^/Li). Moreover, using the SHSPE, full batteries with a lithium metal anode and an LFP or NCM cathode exhibit good performance. The lithium storage capacities in both cases reach 147.9 mAh∙g^−1^ (Li/SHSPE/LFP battery) and 170.6 mAh∙g^−1^ (Li/SHSPE/NCM battery) at 0.1 C. The extremely low polarization between the charge and discharge plateaus of the Li/SHSPE/LFP battery after 20 cycles at 0.1 C indicate the excellent compatibility and stability between the SHSPE and electrodes.

**Figure 10:**
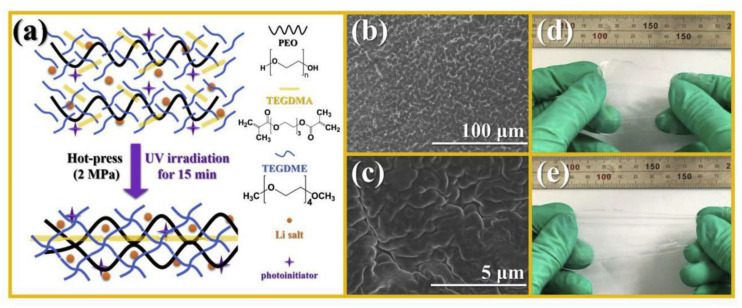
(**a**) Diagram of PTT–SPE inter-crosslinking structure; (**b**,**c**) SEM images of PTT–SPE membrane; (**d**,**e**) Photographs of PTT–SPE (2:1:2) film in stretching mode [97]. Reproduced with permission from [97]. Copyright 2019, Elsevier B.V.

**Figure 11:**
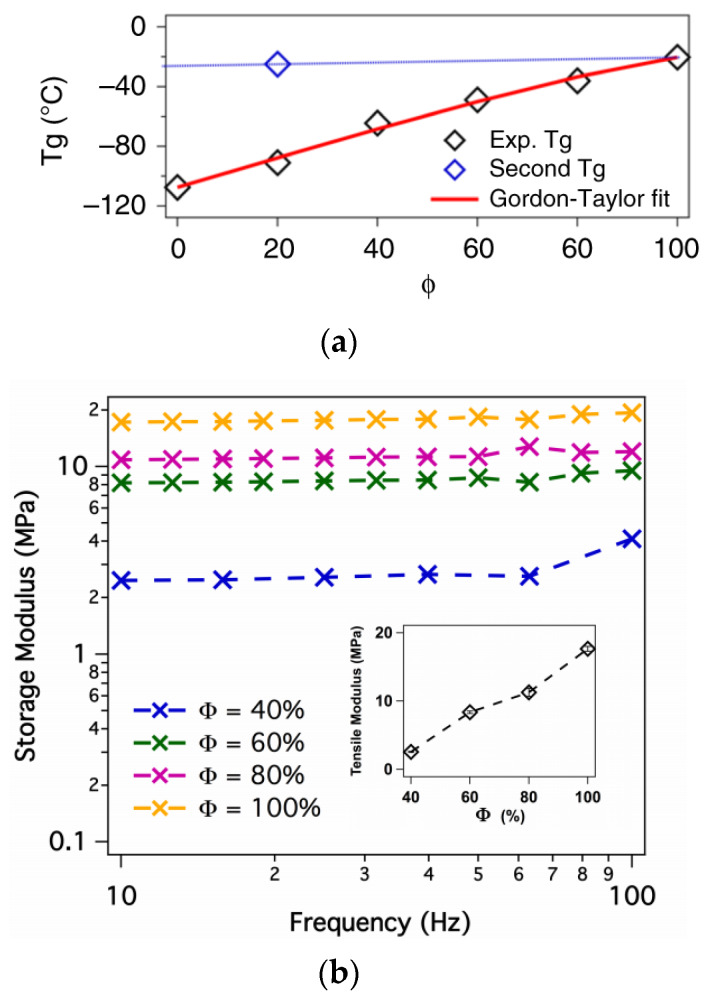
(**a**) Glass transition temperature as a function of poly(ethylene glycol) dimethacrylate (PEGDMA) fraction (Φ) in the membranes. The red line is the Gordon-Taylor fit to the experimental results; (**b**) Tensile analysis of the crosslinked membrane at various PEGDMA content. The oscillatory tensile measurement was done on thin films at a low strain rate of 0.1% in the frequency range of 100–10 Hz. The plateau modulus is plotted as a function of PEGDMA content in the inset [98]. Reproduced with permission from [98]. Copyright 2019, Springer Nature Limited.

Poly(ethylene glycol) dimethacrylate (PEGDMA) and diacrylate (PEGDA) are the most widely used monomers for the chemical crosslinking of polymer electrolytes. The networks are typically prepared by free radical photo-polymerization or thiol-ene reaction. Zhang et al. demonstrated a facile approach by UV irradiation to prepare a flexible PEO-based crosslinked electrolyte (PTT) consisting of linear PEO, tetraglyme (TEGDME), and tetraethylene glycol dimethacrylate (TEGDMA) (Figure 10) [97]. The SPE exhibits superior electrochemical properties with high ionic conductivity (2.7 × 10^−4^ S∙cm^−1^ at 24 °C), high transference number (0.56), wide electrochemical stability window that exceeds 5 V (Li^+^/Li), and low interfacial resistance; similar SPEs have also been reported [98,99]. In the work of Choudhury et al. [98], the crosslinked polymer matrix comprised of PEGDMA and bis(2-methoxyethyl) ether (diglyme), and the *T_g_* and mechanical strength were found to increase with the PEGDMA content as shown in Figure 11. The authors observed that the materials behaved as single-phase soft solids at a critical PEGDMA content of 40%. At this point, the barrier to ionic transport in the oligoether is sufficiently low to produce high ionic conductivity; however, the oligoether is also prevented from exhibiting large-scale convective motions by interacting with network chain segments. At the same time, the authors indicated that the membranes with high crosslinking degree (>40%) are able to completely suppress electroconvective instability. In another report [100], Khurana et al. synthesized a series of cross-linked polyethylene/poly(ethylene oxide) SPEs with high ionic conductivity (>1.0 × 10^−4^ S/cm) at 25 °C and a relatively low storage modulus (G′ ≈ 1.0 × 10^5^ Pa) at 90 °C. In lithium dendrite tests, using symmetric Li/SPE/Li cell, they found that low-modulus cross-linked SPEs exhibit remarkable dendrite growth resistance compared with a linear PEO standard (M_w_ = 900 kg·mol^−1^). This result suggests that a high-modulus SPE is not a requirement for dendrite nucleation and growth. Furthermore, when single-ion segments are incorporated into an SPE network, the transference number improves. Nevertheless, the use of a liquid plasticizer remains advantageous for ion conduction and improving the flexibility of the SPE membrane [101,102].

**Figure 12:**
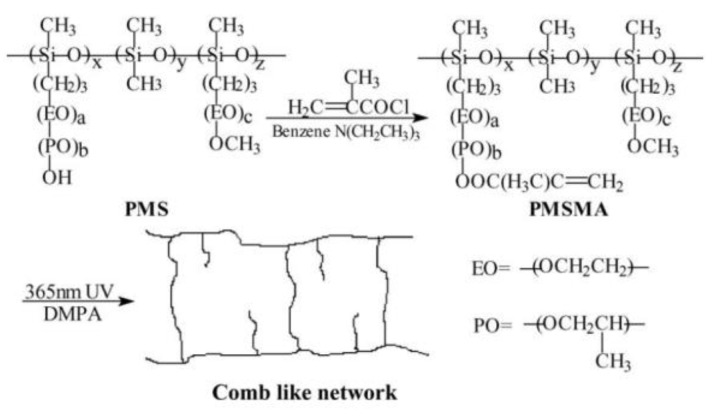
Preparation schemes of comb-like network polymer electrolyte [106]. Reproduced with permission from [103]. Copyright 2005, Elsevier B.V.

In addition to the linear PEO-based networks, crosslinked comb-like polymer electrolytes have also been studied; PEO-grafted polysiloxane is one of the most frequently investigated polymers because of the excellent mechanical and thermal stabilities of siloxane backbone. To prepare this type of network, the polymer chains are commonly crosslinked among themselves or through the addition of a crosslinker. Jiang et al. prepared a comb-like polysiloxane network by photo-polymerization (Figure 12) [103]. They found that the network chain structure has a significant effect on the ionic conductivity and mechanical properties of the electrolyte, and rich PEO side chains enhance conductivity and flexibility. The SPEs exhibit maximum conductivity and tensile strength of 1.01 × 10^−4^ S∙cm^−1^ and 0.66 MPa at 30 °C, respectively.

Oh et al. synthesized a crosslinked mono-comb-type poly(siloxane-*g*-ethylene oxide) electrolyte using PEGDMA as a crosslinker [104]. A maximum ionic conductivity of 1.33 × 10^−4^ S∙cm^−1^ at 25 °C is obtained, indicating that the crosslinking method has no significant effect on the properties of this type of SPEs.

An epoxy group is also employed to prepare a network structure. Daigle et al. prepared a crosslinked SPE composed of a poly(poly(ethylene glycol) methacrylate-*co*-glycidyl methacrylate) copolymer [105]; the polymer chains were crosslinked through amino-epoxy polymerization. The obtained SPE exhibited excellent electric stability and mechanical resistance. Grewal et al. synthesized free-standing polydimethylsiloxane-based crosslinked network solid polymer electrolytes via in situ thiol-epoxy polymerization [106]. In the presence of lithium salt and base catalyst, poly(ethylene glycol) diglycidyl ether, mercapto-terminated polydimethylsiloxane, and pentaerythritol tetrakis (3-mercaptopropionate) were copolymerized at the stoichiometric ratio. Without a liquid plasticizer, the crosslinked SPEs exhibited the following: Ionic conductivities between 1.5 × 10^−6^ S∙cm^−1^ at room temperature and 1.6 × 10^−4^ S∙cm^−1^ at 90 °C; lithium ion transference number of 0.15–0.20; electrochemical window of up to 5.3 V (vs. Li^+^/Li); thermal resistance of approximately 254.5 °C; maximum tensile strength of approximately 1.6 MPa.

A number of SPEs that employ different node groups also exhibit similar electrochemical and mechanical properties. In conclusion, the crosslinking structure affords a stronger mechanical support, and the lithium cation transport in the PEO-based network mainly depends on chain segmental motion and hopping between adjacent coordinating sites. 

### 3.5. Discussion

**Figure 13:**
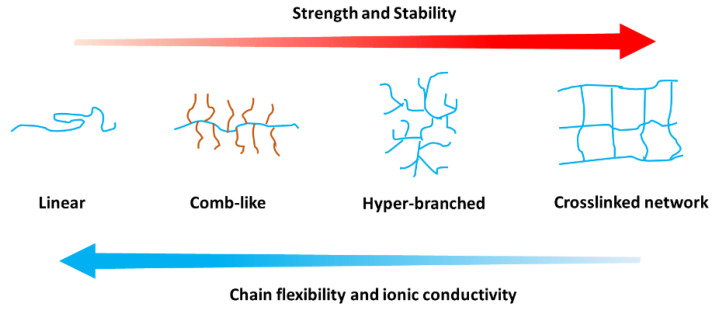
Effect of polymer architecture on electrolyte properties.

After 40 years of development, both the ionic conduction mechanism and practical applications of PEO-based electrolytes have remarkably improved. A significant number of research articles pertaining to PEO-based SPEs have been published, and many factors affecting the performance of this type of SPEs have been investigated. Herein, some effects of polymer architecture on the mechanical and electrochemical properties are summarized (Figure 13), and linear, comb-like, hyper-branched, and crosslinked structures are discussed. Without the addition of any plasticizers, the SPEs with a comb-like or hyper-branched structure exhibit better ionic conductivity at a low temperature compared to that with a linear PEO; the foregoing is attributed to crystallinity reduction or suppression. Moreover, the mechanical strength is enhanced when an amorphous hard backbone is incorporated. With regard to crosslinked SPEs, the crosslinking degree and PEO segment length perform important functions in improving ionic conductivity and mechanical flexibility. In the presence of a liquid additive, the SPEs with a crosslinking structure exhibit excellent ionic conductivity at room temperature as well as good thermal and electrochemical stabilities. 

Furthermore, the incorporation of single-ion segments into these structures can significantly increase the transference number of SPEs from 0.2 to unity. The decomposition mechanism is mainly related to an oxidation reaction of the polymer electrolyte with active materials [107]. The electrochemical stability of SPEs is usually tested by cyclic voltammetry using a symmetric cell with metallic electrodes. Because of the absence of active electrode materials, this measurement method leads to a higher oxidation voltage than that observed in real batteries because of milder test conditions. The decomposition voltage for the PEO-based electrolyte in the presence of active electrode materials is found to be 3.8 V [108], which limits its development in high-voltage ASSLPBs. Besides, it is found that PEO thermodynamic stability on the lithium metal surface mainly relies on its molecular mass [109,110]. A complex architecture often gives a very high molecular mass, therefore, corresponding SPEs exhibit a better stability.

## 4. PIL-Based SPEs

In recent years, the use of ionic liquid (IL) as the ion-conducting medium in lithium batteries has attracted further interest because of its salt solvating capacity, thermal and chemical stabilities. The commonly used cations and anions of ILs have been summarized in several reviews [26,111,112,113,114,115]. Polymeric ionic liquid, sometimes referred to as polymerized ionic liquid or poly(ionic liquid) (PIL), is a macromolecule with ionic liquid units that combines the advantages of ILs and mechanical strength of polymers. In previous years, many of the factors that affect the ion transport in PILs have been investigated. These include the nature of counter-ion [116,117], nature and length of spacer between the main chain and IL moieties [118], and polymer molecular weight [119]. This review is focused on the effects of PIL architecture on the ionic conductivity and electrochemical stability of PIL-based SPEs. Despite the variety of PILs, only imidazolium-based PILs, which are most frequently studied, are considered as an example. 

**Figure 14:**
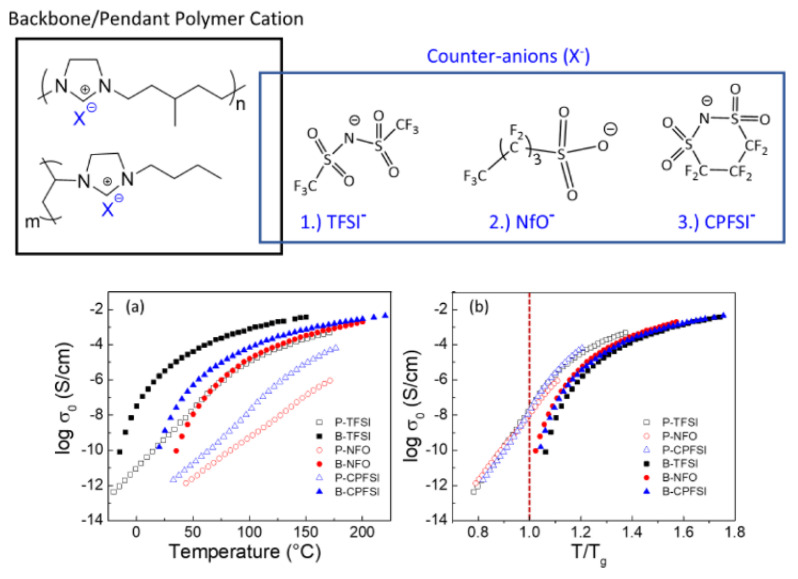
(**Top**) Chemical structures of aliphatic imidazolium-based backbone and pendant PILs and varying counter anions; (**Bottom**) (a) conductivity profiles of B-PILs and P-PILs from DRS shown as a function of temperature (TFSI^−^ = bis(trifluoromethane)sulfonimide anion; NfO^−^ = nonafluorobutanesulfonate anion; CPFSI^−^ = 1,1,2,2,3,3-hexafluoropropane-1,2-disulfonimide anion) and (b) scaled to each material’s corresponding glass transition temperature [120]. Reproduced with permission from [120]. Copyright 2019, American Chemical Society.

Before the properties of PIL-based SPEs are discussed, the effects of architecture on the ion transport in pure PILs are briefly presented. For this type of polymer, IL units can be incorporated into the chain backbone or pendant; their position affects the properties of polymers. Kuray et al. recently investigated the morphology, conductivity, and rheology of imidazolium-based pendant and backbone PILs (P-PIL and B-PIL, respectively) [120]. Figure 14 shows the structure of PILs and their ionic conductivities as a function of temperature. The authors found that B-PILs have a considerably lower *T_g_* than P-PILs with the same counter-anions and also exhibit a higher ionic conductivity on an absolute temperature scale. The conductivity of P-PILs is higher when scaled to *T_g_* or below *T_g_*. This is attributed to the movement of the counter-anions among the side chains in P-PILs (difference in the decoupling degree). Moreover, they found that the ion transport for B-PILs is linked to the segmental motions of polymer chains, whereas that for P-PILs is decoupled from the segmental dynamics at a temperature close to *T_g_*.

Delhorbe et al. systematically investigated the effect of the side alkyl chain length in the imidazolium-based P-PILs on the physicochemical properties and ionic conductivity based on 1-alkyl-3-vinylimidazolium bis(trifluoromethane)sulfonimide-derived homopolymers with various alkyl chain lengths (−C _n_H_2n+1_ (n = 2, 4, 6, 8, 10)) [121]. They revealed that the charge transport in PILs is determined by the segmental motion of the polymer backbone and charge carrier density as well as the self-assembly nanostructure of such PILs, specifically the backbone-to-backbone correlation distance. The low viscosity and *T_g_* favor ion conduction, and the decrease in the backbone-to-backbone correlation distance enhances conductivity. On the other hand, in the presence of LiTFSI with a molar ratio of [Imidazolium^+^]:[Li^+^] = 3:1, they found that the ion transport in PILs also depends on the parameters described above. However, the authors have not identified the lithium cation transport in this system.

The membrane of most PILs is extremely brittle because of insufficient chain flexibility. As a result, the majority of PIL-based SPEs contain a liquid plasticizer, which also aids ion mobility. In a previous study of Hirano et al. [122], the SPE membranes are prepared with a PIL/IL/Li salt ternary system. Poly(1-ethyl-3-vinylimidazolium bis(trifluoromethanesulfonylimide)) (P(EtVIm-TFSI)) is used as the polymer host, and the mixture of 1,2-dimethyl-3-ethoxyethyl imidazolium bis(trifluoromethanesulfonyl) imide (IM(2o2)11TFSI) and LiTFSI is added as the liquid plasticizer. It is found that the ionic conductivity increases with the amount of IM(2o2)11TFSI. With a 65% ionic liquid content, the ionic conductivity reaches 1.89 × 10^−5^ S∙cm^−1^ at 25 °C and 1.84 × 10^−4^ S∙cm^−1^ at 60 °C; however, the mechanical resistance is compromised.

In comparison to linear PILs, the network structure has a higher charge density. Zhang et al. synthesized polymeric ionic networks (PINs) using hexakis(bromomethyl)benzene and 4,4-bipyridine by nucleophilic substitution reaction or a star-shaped imidazolium monomer by free radical polymerization [123]. In the PIN and LiTFSI mixture, it is possible that the crystalline structure of LiTFSI disappears because of the strong electrostatic interaction. Moreover, when the LiTFSI and PINs have the same anion, the LiTFSI ion pair configuration may be restructured. Using PINs as polymer hosts and LiTFSI in 1-ethyl-3-methylimidazolium bis(trifluoromethane)sulfonimide (EMIM-TFSI) (0.5 mol∙kg^−1^) as plasticizer, a six-arm imidazolium monomer-based network SPE exhibits the best ionic conductivity of up to 5.32 × 10^−3^ S∙cm^−1^ at 22 °C and good mechanical properties. Such a high ionic conductivity at room temperature is also observed in other PIL network-based SPEs [124,125]. These results indicate that the network affords higher charge density and better mechanical strength than a linear architecture that enhances the ionic conductivity of PIL-based SPEs. 

## 5. Perspectives

In this review, the effects of polymer architecture on the performance of polymer electrolytes are summarized. With the development of all-solid-state batteries, however, the traditional solid polymer electrolytes (e.g., PEO and polycarbonates) are incapable of satisfying the required high energy density and high voltage. In order to improve the mechanical strength and electrochemical performance of electrolytes, three basic strategies are typically implemented for designing new materials: (1) The use of a highly crosslinked structure; (2) the combination of macromolecules and small molecules (e.g., ionic liquid); (3) the use of organic/inorganic hybrid materials. 

From the perspective of polymer architecture, a highly crosslinked matrix with mobile Li^+^ coordination sites can yield a stable structure to satisfy the demand for high-performance batteries. Indeed, battery performance is a result of electrode and electrolyte cooperation; hence, a good electrolyte design that is suitable for desired electrode materials is necessary.

## Figures and Tables

**Table 1:** Backbone structure and electrochemical properties of comb-like polymers.

Backbone	Chemical Structure	Salt	Conductivity (S∙cm^−1^)	Potential Window (V)	Ref.
Poly(epoxide ether)	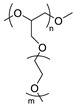	LiTFSI	3.2 × 10^−4^ (60 °C)	ND	[75]
Polysiloxane	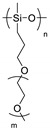	LiTFSI	>10^−4^ (25 °C)	4.5	[63]
Polyphosphazene	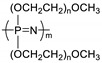	lithium triflate (LiSO_3_CF_3_)	~ 3 × 10^−5^ (25 °C)	ND	[61]
Polymethacrylate	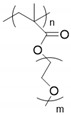	LiTFSI	1.44 × 10^−4^ (30 °C) 7.26 × 10^−4^ (60 °C)	> 5	[65]
Poly(acrylonitrile-butadiene)	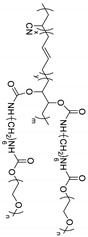	LiSO_3_CF_3_	~3 × 10^−5^ (25 °C)	ND	[68]
Polynorbornene	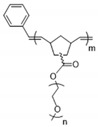	LiTFSI	>3 × 10^−5^ (25 °C) >7 × 10^−4^ (80 °C)	~ 3.5	[67]
Poly(hydroxyl styrene)	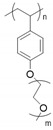	LiSO_3_CF_3_	∼6 × 10^−5^ (60 °C)	ND	[71]
Polyether ether ketone	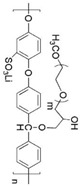	LiClO_4_	> 10^−5^ (room temperature)	ND	[70]
Polypeptoid	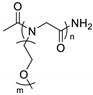	LiTFSI	~10^−5^ (50 °C) 2.6 × 10^−4^ (100 °C)	ND	[69]
Poly(ethylene-co-maleic anhydride)	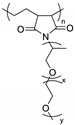	LiTFSI	4.5 × 10^−5^ (room temperature) 5.3 × 10^−4^ (70 °C)	>4 (stainless steel electrode) >3 (aluminum electrode)	[72]

ND = No data
